# Photodynamic Therapy in the Treatment of Cancer—The Selection of Synthetic Photosensitizers

**DOI:** 10.3390/ph17070932

**Published:** 2024-07-11

**Authors:** David Aebisher, Iga Serafin, Katarzyna Batóg-Szczęch, Klaudia Dynarowicz, Ewa Chodurek, Aleksandra Kawczyk-Krupka, Dorota Bartusik-Aebisher

**Affiliations:** 1 Department of Photomedicine and Physical Chemistry, Medical College of the University of Rzeszów, 35-959 Rzeszów, Poland; 2 Students English Division Science Club, Medical College of the University of Rzeszów, 35-959 Rzeszów, Poland; 3 Department of Internal Medicine, Health Care, 39-100 Ropczyce, Poland; kat.bat.szcz@gmail.com; 4 Center for Innovative Research in Medical and Natural Sciences, Medical College of the University of Rzeszów, 35-310 Rzeszów, Poland; kdynarowicz@ur.edu.pl; 5 Department of Biopharmacy, Faculty of Pharmaceutical Sciences in Sosnowiec, Medical University of Silesia in Katowice, Jedności 8 Str., 41-200 Sosnowiec, Poland; echodurek@sum.edu.pl; 6 Center for Laser Diagnostics and Therapy, Department of Internal Medicine, Angiology and Physical Medicine, Medical University of Silesia in Katowice, Batorego 15 Street, 41-902 Bytom, Poland; 7 Department of Biochemistry and General Chemistry, Medical College of the University of Rzeszów, 35-959 Rzeszów, Poland; dbartusikaebisher@ur.edu.pl

**Keywords:** PDT, treatment, synthetic photosensitizers

## Abstract

Photodynamic therapy (PDT) is a promising cancer treatment method that uses photosensitizing (PS) compounds to selectively destroy tumor cells using laser light. This review discusses the main advantages of PDT, such as its low invasiveness, minimal systemic toxicity and low risk of complications. Special attention is paid to photosensitizers obtained by chemical synthesis. Three generations of photosensitizers are presented, starting with the first, based on porphyrins, through the second generation, including modified porphyrins, chlorins, 5-aminolevulinic acid (ALA) and its derivative hexyl aminolevulinate (HAL), to the third generation, which is based on the use of nanotechnology to increase the selectivity of therapy. In addition, current research trends are highlighted, including the search for new photosensitizers that can overcome the limitations of existing therapies, such as heavy-atom-free nonporphyrinoid photosensitizers, antibody–drug conjugates (ADCs) or photosensitizers with a near-infrared (NIR) absorption peak. Finally, the prospects for the development of PDTs are presented, taking into account advances in nanotechnology and biomedical engineering. The references include both older and newer works. In many cases, when writing about a given group of first- or second-generation photosensitizers, older publications are used because the properties of the compounds described therein have not changed over the years. Moreover, older articles provide information that serves as an introduction to a given group of drugs.

## 1. Introduction

Photodynamic therapy (PDT) is an innovative method of cancer therapy that has been developed for several decades and is based on the use of photosensitizing compounds (photosensitizers—PSs), which, after exposure to a laser of an appropriate light wavelength, lead to the destruction of cancer cells using reactive oxygen species (ROS). The main advantages of PDT include its precision, especially in superficial tumors, the possibility of repeating the procedure several times in the same place, the minimal systemic toxicity, and the lack of mechanisms of acquired resistance [1,2,3,4,5,6]. PDT leads to the destruction of the tumor in three ways: I—by direct elimination of cancer cells using reactive oxygen species, II—by affecting the microcirculation of the tumor and depriving it of oxygen and nutrients, and III—by influencing the immune system in order to “set” it to fight cancer cells (including by inducing inflammation in the tumor environment, which causes the release of appropriate chemokines and the influx of immune cells that recognize cancer antigens and “learn” them) [7,8,9,10,11,12]. The universality of PDT’s mechanisms allows it to be used in the treatment of cancers of most organs, especially breast cancer, bladder cancer, lung cancer, head and neck cancer and brain tumors. So far, PDT has mainly been used as a treatment supporting standard methods of therapy (surgery, chemotherapy and radiotherapy), but the continuous development of this technology offers hope for its dissemination in clinical practice as an independent, innovative method of cancer treatment. An ideal photosensitizer for PDT should have the following features: the ability to absorb light waves, preferably in the range of 600–800 nm, high photosensitivity, good biocompatibility, a high molar absorption coefficient, good photostability and affinity for rapidly proliferating cells [13,14,15,16,17]. A very important issue is the selectivity of the photosensitizer accumulation in cancer tissue, so that healthy cells are not damaged during irradiation. To achieve these goals, new technologies are increasingly used to synthesize optimal PSs. There are three main groups of photosensitizers: first generation, which includes, among others, Photofrin—the first PS approved for clinical use, as well as other compounds based on the structure of protoporphyrin—a natural heterocyclic aromatic compound that, thanks to the presence of pyrrole rings, is able to absorb light [15,16,17]. The second generation consists of modified PSs from the first generation and includes macrocyclic compounds based on tetrapyrrole, such as chlorins, bacteriochlorins and phthalocyanines, as well as 5-aminolevulinic acid (ALA) and its derivative—hexyl aminolevulinate (HAL). The second generation of PSs are characterized by a high light absorption coefficient in the near infrared range and an increased ability to generate reactive oxygen species, thanks to which they can be used to treat larger and deeper tumors. Moreover, ALA has been used in intraoperative imaging of brain tumors, thanks to its selective accumulation in tumor cells and its ability to fluoresce, which allows neurosurgeons to perform more accurate tumor resection [17,18,19,20,21]. In turn, HAL is used with good effect to treat bladder cancer [22]. The third generation is based on the second group of photosensitizers, combined with carriers such as monoclonal antibodies, various proteins, carbohydrates or hyaluronic acid or enclosed in nanocapsules (made of gold, silicon or carbon nanotubes). These treatments make the third generation the most selective in combining and accumulating in cancer cells, which leads to the increased effectiveness of therapy while sparing healthy tissues [23,24,25,26,27,28,29,30]. Another important issue is the use of PS nanoparticles for cancer immunotherapy [31,32,33]. Despite significant progress, efforts are still being made to obtain new photosensitizers that will overcome PDT’s barriers, such as limited therapy options for deep and hypoxic tissues [34]. So far, most PSs have been created standardly based on the structure of porphyrins. Other photosensitizer groups include, among others, photosensitizers with absorption peaks in the near infrared range (NIR) such as squaraine, cyanine and croconaine dyes, as well as heavy atom complexes including iridium, ruthenium, iron, iodine or bromine. An interesting group are heavy-atom-free nonporphyrinoid photosensitizers, which include, among others, derivatives of the boron dipyrromethene dye (BODIPY) and modified naphthalimide derivatives in which the oxygen atoms are replaced with sulfur or selenium. Thanks to such changes, PSs enable the production of large amounts of reactive oxygen species even in partial tissue hypoxia, which is a promising step for PDT. Moreover, the combination of the above molecules with the morpholino group causes them to accumulate selectively in the lysosomes of cancer cells, increasing the precision of therapy [6,35,36,37,38,39,40,41,42]. This review collects information about the application of photodynamic therapy in the treatment of cancer, with a particular focus on advances in the creation of novel photosensitizers obtained by synthesis. Thanks to developments in biochemistry, biomedical engineering and nanomedicine, it is possible to produce ever more perfect compounds, so that photodynamic therapy continues to produce better results, directly influencing patient prognosis. This article is based on research from PubMed and Google Scholar on the use of PDT (photodynamic therapy) in the treatment of cancer, with a particular emphasis on the role of synthetic photosensitizers. We searched phrases like “PDT in cancer”, “Synthetic photosensitizers”, “Photosensitizers generations” and “PDT development”. The collected articles were published between 1984 and 2024. A total of 76 out of 143 cited papers were published in the last 5 years (2019–2024). Older ones were mainly used to describe the process of development of synthetic photosensitizers over the years and for the introduction of the PS generations. The article selection was based on title, abstract, language (English and Polish) and availability. Duplicate records were removed. We included reviews and research articles, as well as case reports. The keywords were photodynamic therapy; generation of photosensitizers; and synthetic photosensitizers. Both general information about PDT and the division into the most important groups of photosensitizers are included, as well as examples of new compounds obtained by individual research groups. The aim of the article was to collect information about synthetic photosensitizers—the topic is extremely extensive. The topic was presented in an optimal way, touching on more general issues as well as referring to individual substances and developing more interesting issues. Several PDT review articles are available in the literature, providing different approaches to this therapy, with different perspectives.

## 2. Application of Photodynamic Therapy in Cancer Therapy

Photodynamic therapy is a relatively new method of cancer treatment, which, due to its low invasiveness and high precision, is becoming a promising alternative to traditional methods of tumor therapy. Currently, the universality of PDT allows it to be used in the treatment of cancers of most organs, especially breast cancer, bladder cancer, lung cancer, head and neck cancer and brain tumors [15,16,17,43]. The use of PDT in the treatment of bladder cancer was first approved in Canada in 1994, and since then, scientists have been constantly working on its improvement, including through the synthesis of new photosensitizers [15]. The first such compound was Photofrin, which was adopted by the US Food and Drug Administration (FDA) in 1995 [16]. The essence of photodynamic therapy is the selective destruction of cancer cells through the production of reactive oxygen species, which is achieved by the reaction of a photosensitizing substance settling in the diseased tissue with laser light of an appropriate wavelength. The photosensitizer is most often administered intravenously, thanks to which it quickly reaches the target tissue—this is possible by using receptors present on cancer cells and absent or present in reduced amounts on healthy cells. This function is fulfilled by receptors for LDL plasma lipoproteins, which are responsible for transporting cholesterol needed for the synthesis of cell membranes to cells with a rapid rate of division—in this case, cancer cells. Photosensitizer molecules have a high affinity for LDL, so after combining with them in the plasma, they reach the target tumor tissue [17,18,44,45]. The laser is used to irradiate a specific region of the body where the tumor is located. Various light wavelengths are used for this purpose, depending on the photosensitizer and its absorption range, as well as on the desired depth of penetration of the rays into the tissue. The general range of wavelengths is from approximately 405 to 900 nm, while the most desirable absorption range of the photosensitizer is 600–800 nm [16,46]. After exposure, the photosensitizer is activated, thanks to which energy is transferred from light to molecular oxygen and reactive oxygen species (ROS) are created. This process takes place as follows: after absorbing photons, the photosensitizer moves from the basic energy level (singlet state) to the longer-lasting excited state (triplet). Thanks to this, two types of reactions can occur. The first process involves the direct interaction of the excited triplet with a target substrate, such as a cell membrane or another molecule. As a result, a hydrogen atom or an electron is released, which results in the formation of reactive oxygen species capable of oxidizing specific cellular structures [46,47]. The second reaction involves transferring energy through the triplet state directly to the oxygen molecule, which leads to the formation of highly reactive singlet oxygen. Both of these reactions occur simultaneously, but it is generally believed that the second mechanism dominates and is crucial for the effectiveness of the therapy [48,49]. The result of the reaction depends, among others, on the concentration of the substrate and oxygen, the pH of the environment or the chemical composition of the photosensitizer. PDT of deeper and hypoxic tumors is more difficult due to the low oxygen concentration and limited light penetration into the tissue [46,47,48,49,50,51,52]. As far as photosensitizers are concerned, they can be divided into three groups. The first generation are natural porphyrins and their derivatives, while the second are derivatives of chlorins and phthalocyanines, which are characterized by greater absorption of red light than the first generation and therefore its better penetration into tissues. In turn, the third generation consists of photosensitizer molecules combined with various types of carriers, thanks to which this group is characterized by the greatest specificity in binding to cancer cells. For this purpose, the following are used, among others: antibodies and various proteins, as well as gold, silica and carbon nanotubes nanoparticles [16,53,54]. PDT destroys the tumor in three main ways: by direct destruction of cells using reactive oxygen species, by destroying the tumor microcirculation and by stimulating the immune system. Depending on the intensity of the light administered, cancer cells die by apoptosis (at lower doses of radiation) or necrosis (at higher doses) [7,8,9]. In the case of necrosis, the cell membrane is disrupted and the cell contents are released, which leads to the disruption of local homeostasis and the influx of immune cells. Macrophages engulf damaged cells and present cancer antigens to CD4+ lymphocytes, which in turn stimulate cytotoxic CD8+ lymphocytes capable of directly destroying cells—in this way, the immune system is able to recognize the cancer cell and destroy it [9,10,11]. Photosensitizers accumulate not only in the tumor cells but also in the endothelium lining the microcirculation vessels, which are responsible for its nutrition and providing oxygen. For this reason, when these vessels are destroyed after radiation, the tumor does not receive appropriate substrates for survival and dies [7,8,9,10,11]. The main advantages of PDT include its high selectivity (destruction of tumor cells while sparing healthy tissue), low degree of invasiveness, the possibility of repeating the therapy several times in the same place, fewer possible side effects than in the case of other forms of cancer treatment (minimal skin damage, quick healing and most often leaving no scars), as well as relatively low costs. The limitations of this method primarily include the lack of effectiveness in the treatment of cancers with numerous metastases due to the high precision of the method related to the need for point irradiation and the limited effectiveness in the treatment of hypoxic tumors (e.g., those surrounded by necrotic tissue or very large), as well as those located very deep, due to poor light penetration and the need for surgical intervention to visualize the target area. Irradiation may also cause a photoallergic skin reaction [15,16,17,55]. Despite these difficulties, photodynamic therapy is a promising and future-proof method of cancer treatment that may revolutionize current therapy, allowing for longer survival and increasing patient comfort.

## 3. Photosensitizers in PDT

To date, most clinically approved photosensitizers (PSs) for cancer treatment have been designed based on the structure of protoporphyrin. Porphyrins are heterocyclic aromatic compounds that contain four pyrrole rings connected by methine bridges [5,6]. These are compounds naturally occurring in hemoglobin (iron porphyrin), hemocyanin (copper porphyrin) in animals, as well as in chlorophyll (magnesium porphyrin) and vitamin B12 (cobalt porphyrin) in plants. They intensively absorb electromagnetic radiation—their absorption band is divided into the very intense so-called Soret band in the near ultraviolet (at about 400 nm) and the four less intense so-called Q bands in the visible range (500–700 nm). It is mainly this property that allows the use of porphyrins and their derivatives to create PSs used in PDT. Other beneficial features of these compounds are the high photosensitivity, good biocompatibility, high molar absorption coefficient, good stability and affinity for rapidly proliferating cells [1,2,3,4,5,6]. The first clinically approved photosensitizer was Photofrin, which is a sodium porfimer and a derivative of hematoporphyrin (HpD). This compound absorbs red light with a wavelength of 630 nm, so after irradiation of cancer tissue in contact with oxygen, it allows the formation of highly reactive singlet oxygen, which oxidizes cell components, leading to their destruction [19]. In one example, a laser with a power of 5 mW cm^−2^ of light at a wavelength of 633 nm was used at regular intervals for 5 or 10 min, resulting in a light fluence of 1.5 and 3.0 J cm^−2^, respectively. Photofrin is mainly used in the treatment of esophageal, lung and bronchial cancers. In turn, porphyrin PSs approved for clinical use include Ameluz (basal cell carcinoma of the skin and actinic keratosis), Hexvix (bladder cancer), and Metvix (basal cell carcinoma, Bowen’s disease and actinic keratosis), which are second-generation photosensitizers [16]. Currently, much research is focused on modifying porphyrin-based compounds to achieve better results in PDT. The ideal photosensitizer should be characterized by the highest possible affinity for tumor cells, allowing for precise accumulation in the diseased tissue, low activity and toxicity in the absence of light, high production of singlet oxygen, a light absorption peak between 600 nm and 800 nm, and a rapid elimination rate from the body (depending on the photosensitizer used and the condition being treated, the total light dose should not exceed 200 J/cm^2^) [1,15,16,17,56]. To achieve these goals, subsequent generations of PSs were created, being derivatives of porphyrin compounds or obtained separately by chemical syntheses. Table 1 shows the generations of synthetic photosensitizers.

## 4. Second Generation of Photosensitizers

In order to improve the effectiveness of first-generation photosensitizers used in PDT, other macrocyclic photosensitizing compounds based on tetrapyrrole were created, such as chlorins, bacteriochlorins and phthalocyanines, with a high absorption coefficient in the near infrared range and an increased ability to generate reactive oxygen species. Thanks to this, the new PSs allowed the use of PDT in the treatment of larger and deeper tumors. However, of the thousands of substances obtained, only a small number have been approved by the US Food and Drug Administration (FDA) for use in the clinical treatment of cancer [6]. The approved second-generation photosensitizers include chlorin derivatives (Foscan—head and neck cancer, Laserphyrin—esophageal cancer, lung cancer and brain tumors), bacteriochlorins (Redaporphiner—bile duct cancer) and phthalocyanines, as well as 5-acid -aminolevulinate (ALA) and its derivative—hexyl aminolevulinate (HAL) [16,17,18], Radachlorin (Bremachlorin, which consists of chlorin e6, chlorin p6 and purpurin), and Pheophorbide (Photochlor). Chlorins are tetrapyrrole macrocycles, which are reduced derivatives of porphyrins. They have the ability to absorb light in the 650 to 750 nm region (at a laser light dose of 1 J/cm^2^ in in vitro studies), both red and blue, and to efficiently produce ROS, an advantage over first-generation PSs. Chlorins are a component of many natural substances, such as chlorophyll and various enzymes, as well as being synthesized by marine microorganisms [72,73,74,75,76]. Chlorin derivatives used for PDT can also be obtained by synthesis. Other porphyrin PSs approved for clinical use include Ameluz (basal cell carcinoma of the skin and actinic keratosis), Hexvix (bladder cancer), and Metvix (basal cell carcinoma, Bowen’s disease and actinic keratosis) [16]. Table 2 shows the chlorin derivatives and their molecular structure. 

## 5. Second Generation of Photosensitizers—5-ALA and HAL

**Figure 1:**
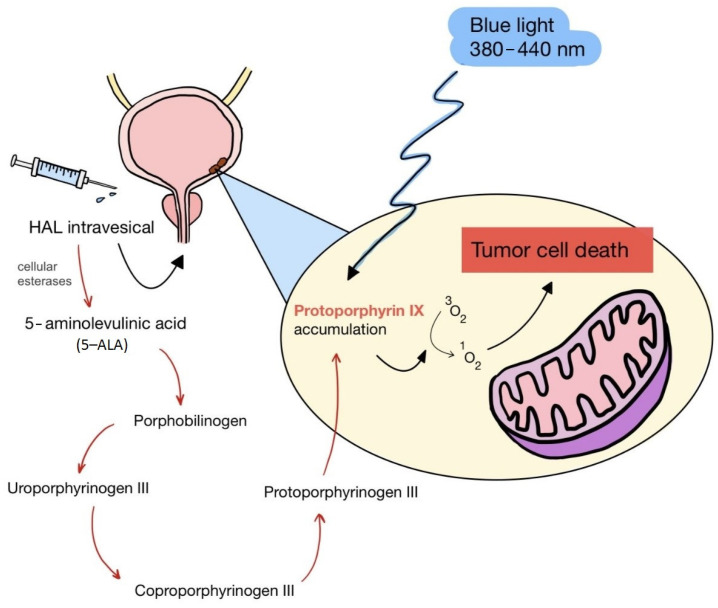
Scheme of action of hexyl aminolevulinate (HAL) in PDT of bladder cancer.

ALA is an organic chemical compound from the group of keto acids and amino acids, a derivative of levulinic acid. It is naturally produced in mitochondria and is a precursor for the synthesis of porphyrins, including heme. After conjugation, eight ALA molecules form protoporphyrin IX (PpIX), which has photosensitizing properties. PpIX has the ability to accumulate in cancer cells, involving the fact of their lower expression of ferrocelatase, which is an enzyme that converts PpIX into heme [18,19,20,21]. PpIX has an absorption peak at 632 nm (red light). The dose of laser light depends on the type of study performed (whether it is an in vitro study, an animal study, or an in vivo clinical study). In one of the in vitro studies, the radiation power was estimated to be 12.4 mW/cm^2^, 11 J/cm^2^, while in animal studies, the dose was 200 J/cm^2^ (100 mW/cm^2^) [20]. ALA is particularly used in the treatment of brain tumors due to its fluorescent properties, enabling better tumor imaging during surgical resections—it is the first agent with such an application approved by the FDA for clinical use [17,20]. In addition, 5-ALA has found use in the treatment of actinic keratosis (AK), where the overall efficacy of the therapy in removing lesions has been estimated at around 80%. In turn, HAL (hexyl aminolevulinate), under the trade name Hexvix, is primarily used in the treatment of bladder cancer. It induces the accumulation of PpIX in cancer cells, thanks to which, when illuminated with blue light with a wavelength of 380–440 nm, the cancer tissue becomes clearly demarcated with red fluorescence. During irradiation, the radiation intensity was set at 7 mW/cm^2^ and the total light dose was 7.5 J/cm^2^ [22]. This is primarily important during surgical tumor resections—more precise removal of the tumor mass improves both the short- and long-term recurrence rates, which translates into longer patient survival [18,22,80]. It is significant that the use of HAL allows the achievement of up to 50 times higher concentrations of PpIX in tumor tissue than when using 5-ALA. This may be due to the greater lipophilicity and amphiphilicity of esters, which allows for easier transport and diffusion through the cell membrane into the cytosol, while ALA requires active transport [81,82,83]. The study by Lamy, L. et al. [22] showed promising results after the use of HAL and blue light cystoscopy in a rat bladder cancer model (Figure 1). A positive antitumor effect was observed in 63% and 31% of rats 12 and 30 days after surgery, respectively. It was also found that the destruction of cancer cells is most likely related to the stimulation of the immune system, as evidenced by the influx of CD3 + and CD8 + T cells into the tumor. Moreover, additional intravesical administration of anti-PD-L1 antibodies resulted in even greater treatment effectiveness—the anticancer result after 30 days increased from 31% to 38%. In turn, the study by Drăgoescu O. et al. [84] emphasized the effectiveness of cystoscopy with HAL in the diagnosis of bladder cancer—this examination allowed the detection of 25.8% more tumors than classic cystoscopy. Early detection of these changes allowed more effective therapy, resulting in a reduction in the number of relapses and an extension of the survival time. The limitations of this photosensitizer group include the still poor water solubility, high aggregation capacity and interaction with proteins and other molecules [85]. These problems were addressed by designing further PS groups. 

## 6. Second-Generation Photosensitizers—Porphyrin Derivatives: Examples from the Literature

Research groups around the world are constantly improving the structure of photosensitizers in order to improve their operation and the effectiveness of PDT. Thanks to these efforts, photodynamic therapy is becoming an increasingly effective and innovative method of cancer treatment, which directly translates into the length of survival and comfort of patients. 

In work by Costa L.D. et al. [2], the researchers presented a new compound: 5,10,15,20-tetra(quinolin-2-yl)porphyrin (2-TQP), created by adding a quinoline group to the porphyrin macrocycle. In this way, in addition to the known photophysical properties of porphyrins, anticancer, anti-inflammatory, bactericidal and antimalarial effects were also obtained. The study showed that 2-TQP is characterized by the ability to more effectively synthesize singlet oxygen and significant in vitro phototoxicity toward HT29 human colon adenocarcinoma cells (a halogen lamp was used, emitting light with a wavelength not longer than 570 nm, with a fluence coefficient of 10.8 mW/cm^2^ or 6.3 mW/cm^2^ depending on the presence of a filter). In this way, it was proven that the newly obtained compound meets two critical requirements for a good photosensitizer in PDT. 

Another study by Mahajan P.G. et al. [3] assessed the suitability for PDT of tetrakis(4-carboxyphenyl) porphyrin (TCPP) derivatives, which were synthesized using IR spectroscopy, proton NMR and mass spectroscopy. The fluorescence quantum yield of the new compounds and singlet oxygen quantum yield, as well as cytotoxicity against two cancer cell lines: MBA-MD-231 (breast cancer) and A375 (melanoma), were examined. At a concentration of 1 μM using two of the synthesized TCPP derivatives, a toxic effect on the tested cancer cells was observed. The obtained results indicated that new porphyrin photosensitizers related to alkylamine and alkylhydrazide may be useful as photosensitizers in cancer therapy using PDT. 

Similarly, in work by Feng X. et al. [4], the properties of two new porphyrin derivatives were presented and tested: P1—2(4)-(1-hexyloxyethyl)-6,7-bis [2-(sodium carbonate)ethyl]-1,3,5,8-tetramethyl-4(2)-vinylporphyrins and P2—4-(1-hexyloxy-methyl)-6,7-bis[2-(sodium)ethyl carbonate]-1,3,5,8-tetramethyl-2-vinylporphyrins. Both compounds showed characteristic absorption spectra and well-defined features by Fourier infrared spectroscopy (FTIR), as well as the corresponding 1 H nuclear magnetic resonance (NMR) spectra and mass spectrometry. More importantly, P1 and P2 are characterized by high phototoxicity at low light doses (under 0.5, 1, and 2 J/cm^2^) toward breast cancer cells both in vitro and in vivo. For these reasons, these newly synthesized porphyrin derivatives represent a promising element for use in PDT.

Another study by Kogan E.A. et al. [86] tested the cytotoxicity of polycationic derivatives of synthetic bacteriochlorin against Lewis lung cancer cells in vitro and in vivo. The results showed that the IC50 values (half maximal inhibitory concentration) for tetracationic PSs were approximately 0.8 μM and for octacation PSs were 0.5 μM. In turn, an in vivo study conducted on mice showed that the tested compounds inhibited tumor growth and extended the animals’ lifespan by over 130%, and they also increased the overall survival by over 50%. Polycationic PSs have been shown to be much more effective in destroying cancer stem cells by inducing necrosis and apoptosis and causing a sharp decrease in mitotic and proliferative activity. Moreover, they are characterized by a greater ability to destroy newly formed tumor microcirculation vessels compared to anionic photosensitizers and ensure the blood flow in the tumor is stopped without hemorrhages and thrombosis.

In the study by Boscencu R. et al. [87], three unsymmetrical meso-tetrasubstituted phenyl porphyrins were designed for photodynamic therapy of cancer: 5-(4-hydroxy-3-methoxyphenyl)-10,15,20-tris-(4-acetoxy-3-methoxyphenyl)porphyrin (P2.2), Zn(II)-5-(4-hydroxy-3-methoxyphenyl)-10,15,20-tris-(4-acetoxy-3-methoxyphenyl)porphyrin (Zn(II)2.2) and Cu(II)-5-(4-hydroxy-3-methoxyphenyl)-10,15,20-tris-(4-acetoxy-3-methoxyphenyl)porphyrin (Cu(II)2.2). Then, the properties important for PDT were tested, such as the ability to produce singlet oxygen, accumulation in cancer tissue and lack of cytotoxicity to healthy cells. The results showed P2.2 to be a promising therapeutic agent for PDT in solid tumors because it generated an acceptable amount of singlet oxygen, accumulated in tumor cells and less in blood cells, showed good fluorescence in cells, allowing tumor visualization, and no significant cytotoxicity in vitro toward healthy cells. For these reasons, this compound has potential for use in PDT of human cancers.

In the study by Wang X. et al. [88], 5,10,15,20-tetra-(phenoxy-3-carbonyl-1-amino-naphthyl)-porphyrin was obtained as a result of the isocyanate condensation reaction, and then its photostability, ability to produce singlet oxygen, and also its cytotoxic effects on human colorectal adenocarcinoma cells were tested. As a result, the new porphyrin showed optimal properties as a photosensitizer in photodynamic therapy, with an in vitro IC50 value of 6.80 μg ml^−1^ after 24 h of incubation. This study indicates the potential benefits of isocyanate condensation in the process of creating photosensitizers.

In the last cited work, Laville I. et al. [89] analyzed the properties of three glycoconjugated porphyrins: TPP(p-Deg-O-alpha-GalOH) (**1**), TPP(p-Deg-O-beta-GalOH) (**2**), TPP(p-Deg-O-alpha-ManOH) (**3**) and their S-analogues in the context of use in PDT of hereditary retinoblastoma. The structure of these compounds was based on the porphyrin core system and the sugar part, which were connected by a diethylene glycol (Deg) bridge. Then, their uptake and photoactivity in retinoblastoma cells were examined in vitro. After preincubation with the appropriate glycosylated albumin, the uptake of (**2**) and (**3**) was inhibited by 40–45%, indicating possible saturation of the cell’s sugar receptor. In turn, compounds (**1**) and (**3**) showed high photoactivity at a light wavelength of 514 nm (5 J/cm^2^), with low metabolic cleavage of O-glycoconjugates and good stability of S-glycoside porphyrins, when examined by mass spectrometry. Based on these results, compounds (**1**) and (**3**) were qualified for in vivo testing.

## 7. Other Second-Generation Photosensitizers with Absorption Peaks in the Near-Infrared Range (NIR)

This group of photosensitizers includes squaraine dyes, cyanine dyes and croconaine dyes. They are characterized by a broad absorption spectral range with high extinction coefficients and high fluorescence quantum yields (QY). In addition, their structure can be easily modified to achieve the desired photophysical properties. They have an absorption peak in the 600–800 nm range (near-infrared (NIR) range), as well as a high singlet oxygen quantum yield, which translates into efficient ROS production upon activation by the laser. In addition, they are rapidly removed from the body, do not accumulate in cells and are not toxic to healthy tissues when not irradiated—so they do not cause many side effects. Among the main disadvantages of this group of PSs are their poor water solubility and limited selectivity for attachment to cancer cells. For these reasons, the use of nanocapsules for transport to the target site with all the beneficial photosensitizing properties of the photosensitizer is being worked on [90,91,92].

**Figure 2:**
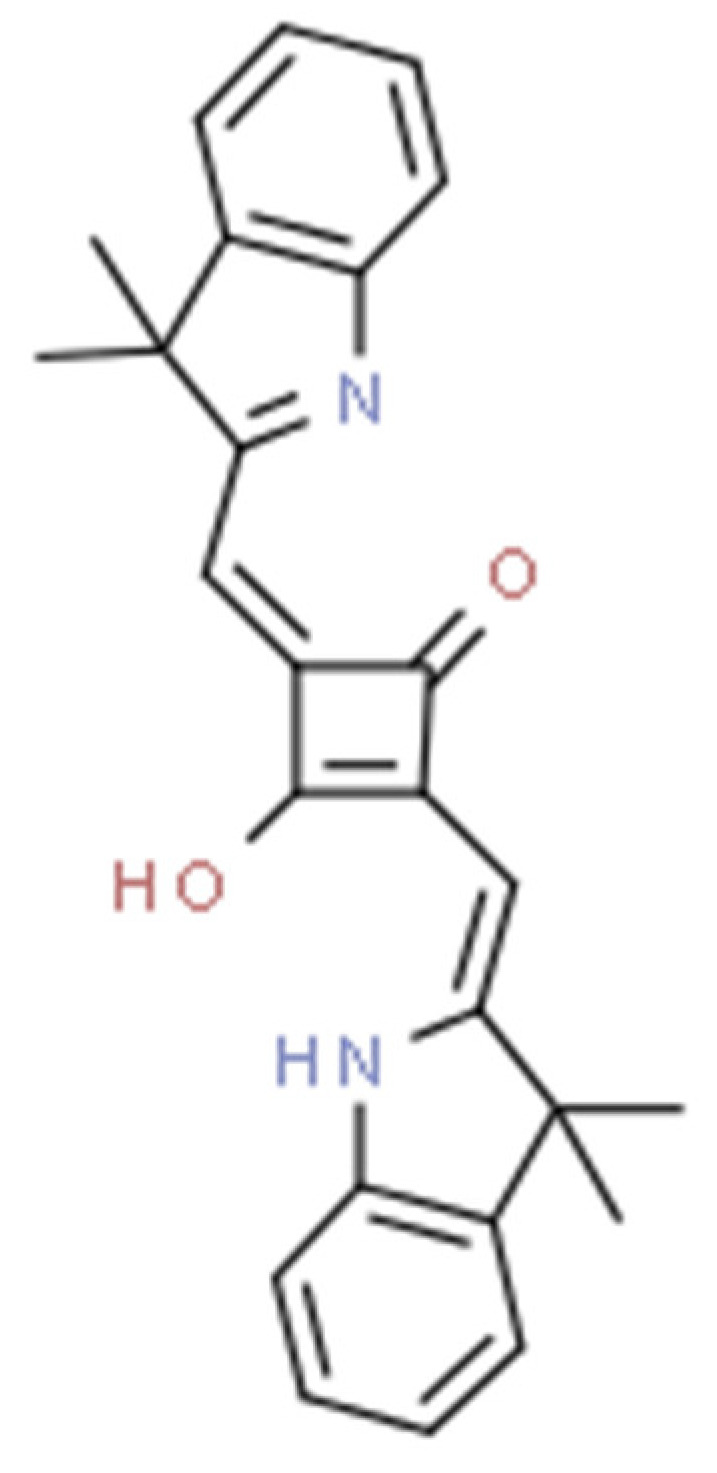
The 2,4-bis[(E)-(1-ethyl-3,3-dimethyl-indol-2-ylidene)methyl]cyclobutane-1,3-dione dye with a squaraine scaffold.

Squaraine dyes consist of four-membered ring systems and are derivatives of squaric acid (Figure 2). They exhibit good photo- and thermostability, as well as fluorescent properties in organic solvents. Unfortunately, in polar solvents, these properties are limited by the aggregation of the dye [90,92].

**Figure 3:**
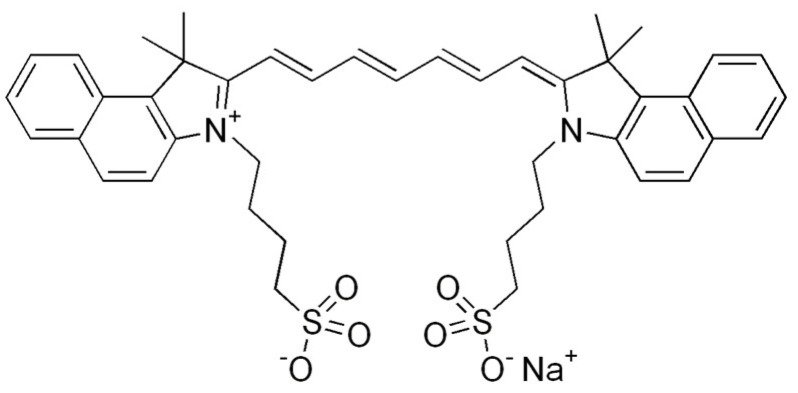
Indocyanine green, a cyanine dye.

Cyanine dyes have similar properties. They are methine dyes, having in their structure two heterocyclic nitrogen rings, which are linked by a π-conjugated chain (Figure 3). Based on the number of methine bonds in the chain, cyanines are divided into monomethine, trimethine, pentamethine and heptamethine. Their structure can be easily modified by halogenation, the attachment of metal atoms or organic structures or conjugation with other molecules, which provides them with new properties [90,93].

Finally, croconaine dyes are pseudo-oxo-carbon compounds obtained from 4,5-dihydroxy-4-cyclopentene-1,2,3-trione. Their structure can by symmetrical or asymmetrical. These compounds are also used in cancer diagnostics—suitable functional groups (e.g., hydroxyl, carboxyl) and markers can be easily attached to their structure to enable tumor imaging. It is also possible to encapsulate a photosensitizer in a nanocapsule to precisely deliver it to the affected tissue [91,94].

## 8. Third Generation of Photosensitizers

The third generation of photosensitizers is characterized by the highest affinity and selectivity in attachment to cancer cells due to the combination of molecules of second-generation photosensitizers with various types of carriers: monoclonal antibodies, proteins, carbohydrates or hyaluronic acid [23,24,25,26,27]. To improve the water solubility, hydrophilic groups were incorporated into the structure of PS molecules: HSO_3_^−^, COO^−^ and NR_4_^+^ [85]. Another method is to produce the transporter in the form of polymer micelles or liposomes. PSs can also be enclosed in nanocapsules: gold and silicon nanoparticles, quantum transporters or carbon nanotubes, which selectively connect to cancer cells [85]. The properties of metal-based nanoparticles are being explored, using molybdenum, titanium, zinc, tungsten oxide and others for their synthesis. Nanocapsules, for instance, allow drugs to be delivered in liquid form. A variety of substances can be deposited on their surface: drugs, prodrugs, contrast agents and various polymers, which allow selective attachment to target cells [85]. Third-generation photosensitizers are currently being tested in preclinical and clinical studies [16,95]. Another important issue is the use of PS nanoparticles for cancer immunotherapy. Thanks to new intelligent nanotechnologies, it is possible to influence the pharmacokinetics of PSs—preventing premature transfer of PSs from tumor cells to the peripheral blood, as well as avoiding the ACQ (aggregation-caused quenching) effect in the tumor environment. The other thing is observing in vivo the distribution of PSs in the system in order to achieve appropriate accumulation of the compound in cancer tissue. Furthermore, it is possible to deliver oxygen to hypoxic tumors, which is crucial for the success of PDT. The use of nanotechnology in PDT increases the effectiveness of immunotherapy by stimulating immune cells to eliminate cancer cells [31,32,96,97,98].

## 9. Evaluation of the Efficacy of Third-Generation Photosensitizers—Examples from the Literature

In the study by Chen Z. et al. [66], a hybrid of a photosensitizer molecule with the protein hemoglobin and human plasma albumin was obtained in order to supply cancer cells with the oxygen needed for the photodynamic reaction to occur. In addition to increasing the effectiveness of PDT, immune stimulation was also achieved through the influx of CD 8+ lymphocytes into the tumor environment, as well as increasing the release of damage-associated molecular patterns (DAMPs) to activate more dendritic cells (DCs), NK cells and CTLs.

**Figure 4:**
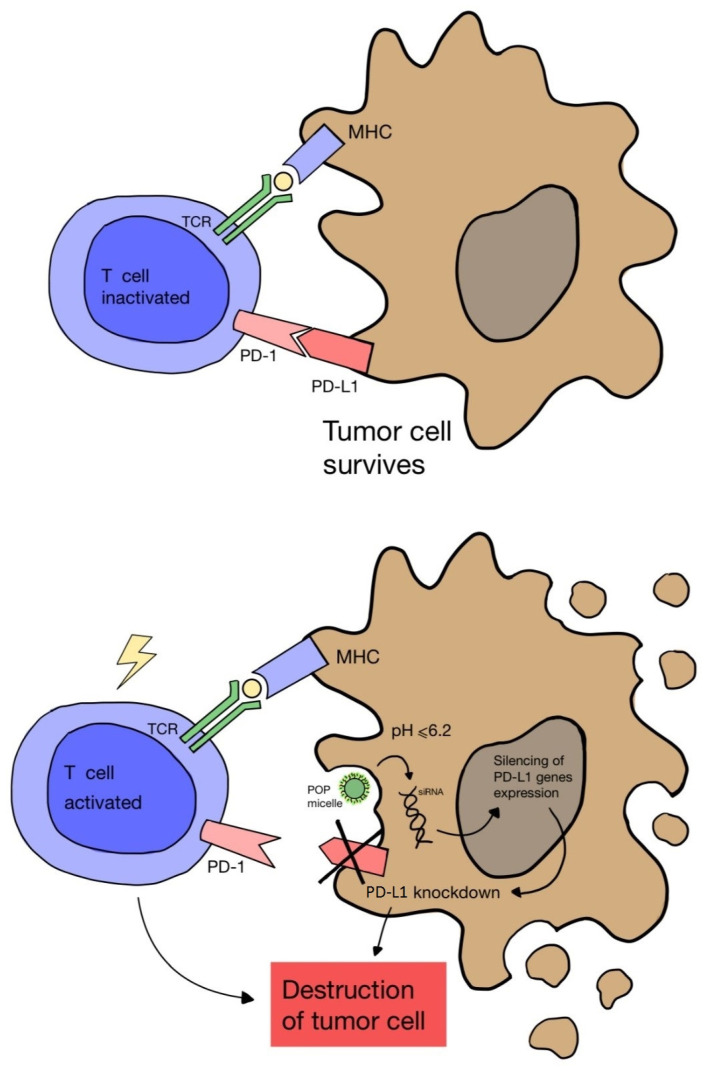
Scheme of blocking the expression of the PD-L1 ligand on a cancer cell using nanomicelles combined with siRNA and a photosensitizer, which are activated in the acidic environment of the cell. Failure to combine PD-1 on the T cell surface with PD-L1 on the cancer cell leads to lymphocyte activation and cancer cell death.

Another study by Wang D. et al. [67] proved that cancer immunotherapy via PDT can be enhanced by blocking the expression of the PD-L1 ligand on cancer cells. A nanocomplex consisting of a cationic micelle activated in an acidic environment, a photosensitizer and small interfering RNA (siRNA) was designed. The complex was only activated in the acidic endocytic vesicles of cancer cells, which enabled fluorescence and PDT imaging. As a result, the combination of PDT and PD-L1 blockade showed greater effectiveness in inhibiting tumor growth and preventing distant metastasis in a melanoma model (Figure 4).

In turn, the study by Ai X. et al. [68] addressed the problem of the limited penetration of PSs into tissues. A unique nanoconjugate of UCNs (a nanomaterial capable of converting light from low to high energy through sequential excitation with multiple photons via an anti-Stokes emission process) with a photosensitizer was developed by integrating manganese dioxide (MnO_2_) nanoparticles and hyaluronic acid (HA) biopolymer to improve the effectiveness of PDT by tissue oxygenation and synergistic reprogramming of the tumor microenvironment. After reacting with H_2_O_2_ produced in the acidic tumor environment, the MnO_2_ nanoparticles were degraded to produce a large amount of oxygen, which significantly increased the effectiveness of PDT after irradiation with near-infrared (NIR) light at a wavelength of 808 nm (0.4 W/cm^2^). Equally important, the HA polymer can effectively reprogram the polarization of pro-tumor M2-type TAM macrophages to antitumor M1-type macrophages to prevent tumor recurrence after PDT treatment.

Similarly, in the study by Cui S. et al. [99], scientists developed a nanoconstruct consisting of UCN nanoparticles and a photosensitizer—zinc (II) phthalocyanine (ZnPc). After its use in a study on small animals, the increased selectivity of the carriers toward cancer cells overexpressing the folate receptor was found. In vivo PDT treatments on deep-seated tumors showed that NIR light-triggered PDT based on nanoconstructs had significant therapeutic efficacy, with a tumor growth inhibition rate of 50%, compared to conventional visible light-activated PDT, with a rate of 18%.

In another work by Lu K. et al. [69], a nanostructure of the organometallic Hf-porphyrin, DBP-UiO, was developed as an exceptionally effective photosensitizer for PDT of head and neck cancer. After its use in an experiment on mice, PDT was significantly more effective both in vitro and in vivo: half of the mice that received a single dose of DBP-UiO with a single exposure to light experienced complete tumor eradication. These results indicate promising prospects for the use of these photosensitizing nanostructures in the treatment of resistant cancers in the clinic.

In turn, the study by Pucelik B. et al. [70] tested the use of pluronic redaporphyrin micelles in the treatment of melanoma. It was found that such a modification of the photosensitizer under in vivo conditions showed increased selectivity toward tumor cells and more efficient cellular uptake, and it led to greater production of reactive oxygen species. As a result, a one-year treatment with this method resulted in a 100% cure of melanoma.

A recently cited study by Ibarra L.E. et al. [71] evaluated a method of delivering polymeric nanoparticles on a monocyte carrier for the treatment of glioblastoma multiforme using human and mouse monocytes. Monocytes loaded with conjugated polymeric nanoparticles were shown to fulfil their role in transporting these compounds into glioma cells—this PDT method was found to be more effective than using photosensitized nanoparticles alone.

Nanotechnologies enable the optimization of the drug biodistribution due to the ability to place a variety of molecules on the carriers’ surface, which adds new biochemical properties. To date, this is the most advanced group of photosensitizers. The above evidence points to the key role of biotechnology and nanomedicine in the development of photodynamic therapy.

## 10. Third Generation of Photosensitizers—Antibody–Drug Conjugates (ADCs)

**Figure 5:**
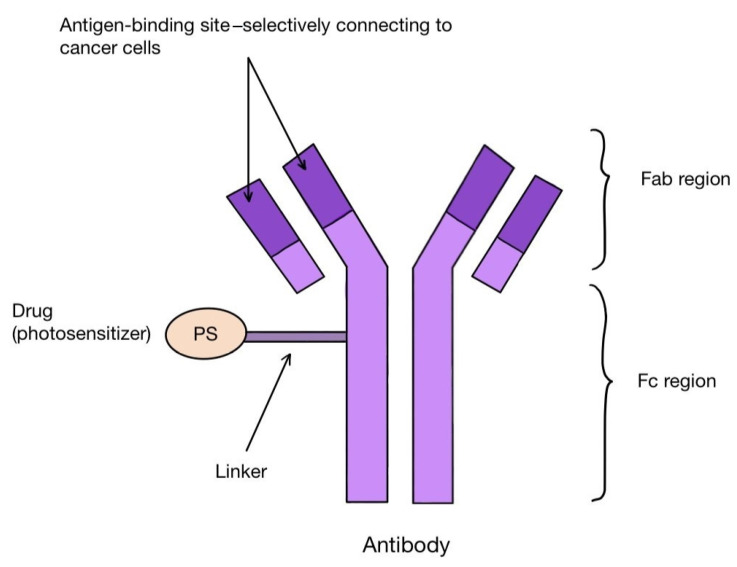
Scheme of an antibody–drug (photosensitizer) conjugate, which can be used for targeted cancer therapy.

One method of creating the third generation of photosensitizers is to combine their molecules with antibodies. These are antibody–drug conjugates (ADCs). The antibodies combine with the corresponding antigens on the tumor cells, allowing the PS molecules to reach their precise destination (Figure 5). Among others, the combination of the photosensitizer BODIPY with trastuzumab, a monoclonal antibody that binds to the HER-2 receptor on the surface of breast cancer cells, is known. Other targets for antibodies can be, for instance, the transmembrane protein CXCR4 present on many cancer cells, the epidermal growth factor receptor (EGFR), folate receptors (FR), prostate specific membrane antigen (PSMA) or CD40 antigen, as well as the estrogen receptor (ER), p-glycoproteins and integrins [100,101,102,103,104,105]. A photosensitizer should have certain characteristics that favor binding to antibodies. These include having a single functional group that reacts with the antibody, solubility in a polar environment and high hydrophilicity [106]. The conjugation itself can take place either directly or indirectly, with as little interference with the structure of the antibody as possible [106]. After the photosensitizer binds to the target cell and is irradiated with the laser, it labels the affected tissue by visible fluorescence or destroys it by reactive oxygen species. In this way, PDT also has a diagnostic function in the early detection of tumor localization. For such use, the name PDD (photodynamic diagnosis) is applied [106]. PS–antibody conjugates thus have a dual role: diagnostic and therapeutic. This approach is described as theranostics. One of the proteins overexpressed on tumor cells is CD142-tissue factor TF. It can be found on the surface of breast cancer cells, including triple negative carcinoma (TNBC), melanoma, lung cancer, ovarian cancer, pancreatic cancer, colorectal cancer, hepatocellular carcinoma or glioma, as well as many leukemic cells [106]. The presence of TF has been linked to the propensity of tumors to grow rapidly, undergo intense angiogenesis and form metastases [107,108]. In addition, it has been discovered on epithelial cells lining newly formed tumor blood vessels as a result of stimulation by VEGF (vascular endothelial growth factor) [101]. In the work by Hu Z. et al. [101], the authors applied fVII (factor VII-ligand for TF, which was appropriately modified by swapping lysine 341 for alanine) in combination with a photosensitizer (fVII-tPDT) to selectively bind to TF located inside melanoma, prostate, head, neck, lung and breast cancers microvessels, as well as in age-related macular degeneration (AMD) and endometriosis. Animal models and phase I and II clinical trials in patients with AMD were used. The results showed efficacy and safety in the treatment of lung and breast cancer, macular degeneration and endometriosis. However, caution should be taken with the systemic use of fVII-tPDT, as VEGF and other cytokines can stimulate TF expression in healthy vessels as well [101].

## 11. Heavy-Atom-Free Nonporphyrinoid Photosensitizers—Group Description

So far, the standard procedure for designing new photosensitizers has been to incorporate heavy atoms into their structure to facilitate the creation of their triplet excited states. Heavy atoms used in this way include, among others, ruthenium, iridium, iodine and bromine, which promote the intersystem crossing (ISC) process through spin-orbit coupling (SOCT) interactions necessary for the transition to the active triplet state [109,110]. However, the above method is associated with certain consequences, including the risk of increased toxicity in the dark, poor photostability of PSs, the short triplet period and the high cost of heavy metals. For these reasons, the use of other alternatives allowing the promotion of ISC is increasingly being considered, e.g., using a spin converter or excitonic coupling, through doubly substituted excited states, rotation of aromatic systems or induction by radicals [111,112,113,114,115,116]. The formation of triplet excited states in electron donor–acceptor systems in the spin–orbit charge-transfer intersystem crossing (SOCT-ISC) process does not require the introduction of heavy atoms into the molecule. Light-induced electron transfer between the donor (D) and the acceptor (A) subunits leads to the formation of a charge transfer (CT) state, which then undergoes nonradiative charge recombination to the ground state (CRs) or to the lowest triplet excited state (CRT) via SOCT-ISC. So far, this phenomenon has been observed in the donor–acceptor systems of advanced intelligent photosensitizers without heavy atoms, such as BODIPY and other difluoroborone complexes, metal dipyrrines, phenoxazines, biphenyls, naphthalene and perylene imides [117,118,119,120,121,122,123]. Heavy-atom-free nonporphyrinoid photosensitizers are a new alternative to classic porphyrin derivatives, and many studies have been conducted on them. Their advantages include the optimal photochemical and photophysical properties for PDT, as well as lower production costs [6].

## 12. BODIPY

**Figure 6:**
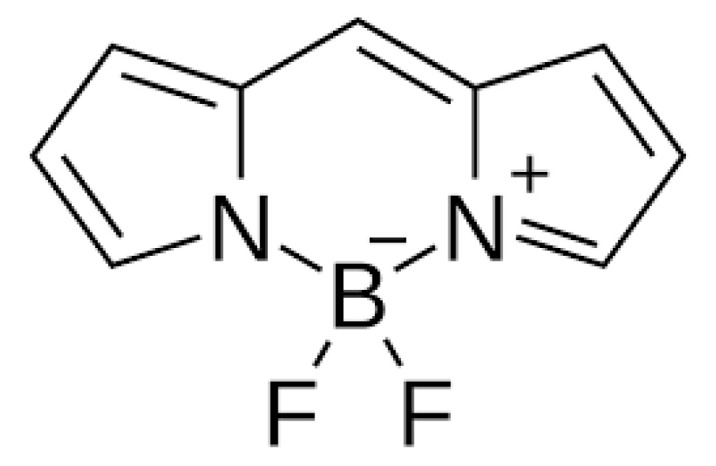
BODIPY [137].

The first photosensitizer mentioned is a boron dipyrromethene dye, specifically 4,4-difluoro-4-bora-3a, 4a-diaza-s-indacene (BODIPY for short (Figure 6)), which was first described in 1968. It has unique features desirable for PDT, such as high visible light extinction coefficients, a high fluorescence quantum yield, tunable absorption/emission from visible to near-infrared light, high photostability and chemical stability, and feasible derivatization [35,36,124,125]. Unfortunately, it is also characterized by the low quantum efficiency of the triplet state, which prevents the efficient production of free oxygen radicals and therefore, initially, heavy atoms had to be included in its structure. Currently, work is being conducted to design BODIPY that would be devoid of them [6,126]. The first such construction of BODIPY was described in its covalent combination with buckminsterfullerene C60, but the practical use of this compound in therapy is not possible due to the complicated and time-consuming synthesis process [127,128]. For this reason, the use of BODIPY was considered in the design of SOCT-ISC photosensitizers due to their favorable photophysical properties.

Various electron donors, such as anthracene, pyrene, perylene, phenothiazine, carbazole and phenoxazine, were used in the tests [129,130,131,132,133,134]. The most important features of BODIPY that enable the use of SOCT-ISC technology are the optimal: (1) mutual orientation of donor and acceptor subunits; (2) values of the driving forces for charge separation and recombination processes (ΔGCT and ΔGCR, respectively); and (3) the ratio between the charge recombination rates to the ground state and to the lowest triplet excited state (kCRS and kCRT, respectively) [126,135]. BODIPY is an interesting photosensitizing compound with prospects for effective use in PDT. Future research should focus on its use in practice, and also in combination with targeted techniques in cancer treatment—conjugation of BODIPY molecules with ligands for receptors that are overexpressed on cancer cells may help to additionally intensify the therapy [136].

## 13. Heavy-Atom-Free Nonporphyrinoid Photosensitizers—Literature Examples

Another group of photosensitizers without heavy atoms are PSs based on biocompatible sulfur-substituted carbonyl fluorophores (in most of the cited articles, modifications are made to naphthalimide derivatives). These compounds are characterized by very good photochemical and photophysical properties, including a high triplet quantum yield, red-shifted absorption maxima and increased absorption coefficients. These features make these substances seem promising for use in PDT, but they have one drawback: they absorb UVA light, which makes them impossible to use for large and deep tumors [37,38,39].

In 2019, a study by Nguyen V.N. et al. [6] was conducted in which the use of sulfur-substituted naphthalimide derivatives in photodynamic anticancer therapy was tested, which showed that these compounds are able to effectively generate ROS. The transition from the singlet state of the photosensitizer to the excited triplet was facilitated by changes in the structure of the naphthalimide derivative molecule: replacing both oxygen atoms with sulfur atoms and introducing electron-donating groups to the 4-position of the naphthalimide platform. The photosensitizer constructed in this way allowed the destruction of cancer cells with only 1% oxygen content in the environment and showed negligible toxicity in the dark, which makes it a suitable candidate for further research in order to introduce it into therapy. The researchers then synthesized another compound by substituting sulfur for the oxygen atoms in the morpholine-substituted naphthalimide derivative, which also led to effective ROS production and cancer cell death. Moreover, thanks to the presence of morpholino groups, this compound is selectively located in the lysosomes of cancer cells, thanks to which it is characterized by the high selectivity of accumulation in target cells, which is desirable from the point of view of PDT.

Similarly, in the study by Pham T.C. et al. [40], a photosensitizer consisting of naphthalimide and pyridine-2(1H)-thione derivatives was synthesized, which can be activated in hypoxia and allows PDT to occur continuously in the light/dark cycle. This is possible due to the presence of the pyridone part, which reacts with singlet oxygen to form endoperoxide. In the absence of light, endoperoxide releases stored singlet oxygen without other side reactions and returns to its original form. Oxygen generated during attempts to use the new PS in PDT induced apoptosis in cancer cell cultures, providing a positive experimental result. Replacing the oxygen atoms in naphthalimide derivatives with selenium may provide similar beneficial effects.

Selenium is an essential element with significant and unique properties, not only chemical but also physical and biological. Jiang et al. [138] conducted research on the design and development of four selenium-based photosensitizers. The main idea was to replace the oxygen atom. As the experiment showed, the designed photosensitizers are stable and can be applied in a non-strongly acidic environment. The authors proved that the concept of substituting heavy atoms in the design of new photosensitizers may be appropriate and contribute to increasing the effectiveness of PDT [138].

## 14. The Potential Problems of PDT

The main challenge related to the application of laser light in PDT in in vivo clinical trials is the depth of the location of cancer lesions qualified for treatment. The maximum penetration depth is one of the main factors affecting the effectiveness of PDT.

PDT usually uses a smaller absorption band of a given photosensitizer, as it is characterized by deeper tissue penetration due to its optical properties, which attenuate light in the tissues intended for treatment. This attenuation of light by the tissues can be attributed to the reflection, absorption, scattering and refraction of light [138]. The effect on the loss of light density due to reflection and refraction depends on the relative values of the refractive indices and is proportional to the angle of incidence of light between the two media. Therefore, their impact can be minimized by lighting perpendicularly in the direction where the two different media meet. However, the scattering of light in tissues has the greatest impact on the attenuation of light intensity as well as its directivity, reducing the depth of light penetration in tissues such as tumors. The light emitted by the laser used in photodynamic therapy (PDT) only penetrates tissues to a certain depth. The optimal optical range in tissues is between 600 and 1200 nm, which allows for the best light penetration. Therefore, most approved and clinically used photosensitizers (PSs) are activated at wavelengths in the 600–800 nm range. However, even when light is used within this therapeutic window, the size of the tumor may exceed the maximum light penetration depth, making the tumor less suitable for PDT. Consequently, the maximum propagation of the treatment light and the locations that can be reached with the light source determine which tumors can be effectively treated with PDT. For this reason, cancers commonly treated with PDT include superficial malignancies or disorders that can be accessed with a flexible light source. In addition to the previously mentioned use of PDT in oncology, it is also used in other medical fields, such as dermatology (actinic keratosis, Bowen’s disease, basal cell carcinoma), ophthalmology (including the treatment of age-related macular degeneration (AMD)), cardiovascular diseases (e.g., esophageal varices), neurology (Alzheimer’s disease) or rheumatology (rheumatoid arthritis), as well as in the treatment of cystic fibrosis or Crohn’s disease [139]. In clinical PDT, the radiation doses that can be applied to the patient are 50 ÷ 200 J/cm^2^, and the radiation density doses are 50–150 mW/cm^2^ for wavelengths of 630, 650 and 670 nm. The exposure time should be 15–20 min and the light source should have a power of at least 1 W. For small surfaces, up to 2 cm^2^, sources with a power of 200–500 mW are sufficient, and for larger surfaces, a light power of 1.5–3 W is necessary [139]. Selection of the appropriate light source is an equally key and important factor determining the effectiveness and efficiency of PDT. Currently there are four sources of light used in clinical PDT: fluorescent and incandescent light, daylight, LED (light-emitting diodes) and laser [140]. The exposure to daylight is only used in dermatology for managing skin lesions like actinic keratosis. It is the cheapest way, because after the PS (mostly 5-ALA derivatives) is applied to the skin, no special source of lighting is necessary [140]. LED-light, especially blue light (wavelength of about 400 nm), is also used in dermatology, mostly for skin lesions (AK) and acne. On the other hand, the red light (630–700 nm) enables deeper penetration and can be considered in oncology, as well as gynecology, otolaryngology and urology [141]. When it comes to the use of PDT in dermatology, some patients do not opt for this form of therapy or give up before completing the treatment. The reasons for this may include fear of side effects, higher treatment costs than anticipated, logistical difficulties in getting to the site of the procedure or observed unsatisfactory results of the therapy [142]. The occurrence of side effects, including their scale and intensity, is also one of the challenges of PDT. The most common side effects of PDT treatment of skin lesions include swelling, redness and pain of the area treated. In addition, hyperpigmentation and fever may occur [143]. The pain or burning sensation usually appears within minutes of the onset of light exposure because of nerve stimulation or tissue damage by reactive oxygen species, possibly exacerbated by overheating. Some patients might benefit from using local anesthetics, such as tetracaine gel, a mixture of lidocaine 2.5% and prilocaine 2.5% or morphine gel [139]. Skin hypersensitivity to light is another aspect related to the potential adverse effects of PDT on the human body [144]. According to Correia et al., the safest photosensitizers are those with the shortest duration and degradation in the human body. These types of photosensitizers minimize skin hypersensitivity to light [16]. This type of PDT effect very often serves as an indicator of the pharmacokinetics of photosensitizers and light sources. When assessing skin hypersensitivity to light, the speed of the photosensitizer dissolution process in the human body is assessed. The first articles that presented the problem of skin hypersensitivity to light after PDT treatment were published as early as 1989. These articles contain recommendations, e.g., to avoid sunlight for 1 month from the date of application of the photosensitizer and light therapy [145]. Currently, doctors recommend staying indoors and avoiding exposure to sunlight for one or two days. It is also recommended to avoid bright lighting indoors due to the skin’s sensitivity to light, which is initially very high. There are also other recommendations, such as wearing appropriate protective clothing immediately after therapy, limiting exposure to sunlight for several days, or using sunscreen. It is important, therefore, that these issues are thoroughly discussed with the physician before treatment begins, to increase patient awareness and to ensure that the patient receives the appropriate form of therapy. How to effectively deliver the light source to the treatment areas is also an important aspect to consider before planning and performing PDT. Lasers are the main source of light used for PDT in clinical indications other than superficial skin lesions. In most clinical situations, the light of the laser is delivered through an optical fiber with a defined power density controlled by the operator. Fiber optics allows either forward delivery or interstitial and intraluminal light emission. In turn, diode (semiconductor) lasers enable suitable light emission by a smaller device and therefore have become more popular [140]. Another aspect is the fluence coefficient and the power output over time to the surface of the treated area [146]. It is assumed that low fluence rates are superior in terms of the antitumor activity compared to high fluence rates in terms of both the antitumor efficacy and inflammatory cytokine production in the tumor area. According to this accepted standard, lasers with a high fluence index will not be as effective. Therefore, selecting the appropriate laser depending on the examination being conducted or the lesion being treated is one of the main factors determining the effectiveness of PDT. The limited general availability of photosensitizers, the lack of adequate training to perform the procedures, and the scarcity of equipment lasers and other light sources still remain challenges for clinicians. It will certainly take some time until PDT becomes a commonly used treatment method for the described conditions, available in most specialized centers. For now, the development and clinical tests of new photosensitizers are the priority. The latest trend in PDT is the search for new concepts for the design of new photosensitizers. The main goal of obtaining new photosensitizers is to overcome previous limitations of PDT. For example, to maximalize the tumor suppression, the organic chromophores are being conjected with metals to enhance the lifetime of oxygen-excited states. To this group belong Ru (II) and Ir (III) complexes. Another way is the synergic work of PDT and chemotherapy by conjugating PSs and chemotherapeutics [147]. For example, oxaliplatin has been used in combination with Verteporfin in the treatment of a metastatic pancreatic cancer cell line with good effectiveness, as well as the oxaliplatin–cucurbit[8]uril complex in combination with acridine orange has shown enhanced tumoral cell-killing ability in vitro [148,149,150]. The other chemotherapeutic is 5-fluorouracil, which in combination with 5-aminolevulinic acid is being tested on squamous cell carcinoma cell lines. Other drugs combined with PSs like Photofrin, 5-aminolevulinic acid or aluminum phthalocyanine include doxorubicin, mytomycin, cisplatin, methotrexate and gemcitabidine. What is more, several chemotherapeutic molecules (cabazitaxe, doxorubicin and irinotecan) have been encapsulated in PoP liposomes, which enhances the selectivity of therapy [151,152]. Another limitation of current PDT is the poor tumour selectivity. The solution is adding peptides or antibodies to the PS molecules, which enhance the targeting ability of drugs [147]. However, it is likely that only a relatively small fraction of injected biologics can finally reach their parenchymal targets, such as 1–10 parts per 100,000 for antibody conjugates and ∼0.7% nanoparticles [153]. To enhance the effectiveness, SIT (structure-inherent targeting)-based PSs were developed. These chemical structures have native targeting potency for tumor cells, which offers a new means of delivery, eliminating the need for conjugations. The main limitation of this group is the time-consuming and inefficient synthesis process. The solution to this problem was suggested in the work by Li M. et al. [153], where the researchers came up with the idea of innovating the strategy for using the Förster resonance energy transfer (FRET) mechanism to achieve SIT features in a single small molecule and significantly amplifying the therapeutic outcome. In turn, the limited penetration depth of many photosensitizers can be changed by extension of the p system of the ligand to red-shift the MLCT absorption, as well as increasing the photon-capturing ability in the NIR region or developing chemiluminescence/bioluminescence-based PSs with high levels of ROS generation [147]. Chemiluminescence PSs are based on the aggregation-induced luminescence (AIE) phenomenon, which means that some fluorescent molecules show weak fluorescence emission in solution and increased fluorescence when they aggregate. AIE PSs are characterized by excellent light stability, end-group modifiability, strong fluorescence characteristics of aggregated states and controllable excitation wavelength, which enhances their deep tissue penetration ability and makes the AIE PSs effective theranostic agents [154]. Some other current challenges in relation to PS development are hypoxic ROS generation systems tolerant of hypoxic conditions, limitation of the oxygen-induced deactivation of excited states, designing PSs with high ISC rates, long T1 lifetimes and therapeutic excitation wavelengths (600–900 nm), as well as producing tumor-microenvironment-activatable PSs and creating strong photon-capturing ability under deep tissue [154].

## 15. Conclusions

The continuous development of technologies enabling the design and synthesis of new photosensitizing compounds is crucial to increasing the effectiveness of photodynamic therapy in oncology. The use of nanomedicine seems particularly promising—by combining PS molecules with various types of carriers and nanocapsules, it is possible not only to reach tumor tissue more precisely and in a more controlled manner but also to overcome current PDT barriers, such as the difficult production of reactive oxygen species in deep and hypoxic tissues, among others, by combining PSs with hemoglobin or through a unique UCN nanoconjugate. In turn, selective targeting of cancer cells may be improved by nanoconstructs such as pluronic redaporphyrin micelles or the combination of UCN with zinc(II) phthalocyanine. In turn, organometallic Hf-porphyrin (DBP-UiO) has been shown to be effective in the treatment of clinically resistant cancers. Research is also ongoing on the construction of photosensitizers that would be devoid of heavy atoms, such as ruthenium, iridium, iodine and bromine, and whose applications include, among others, an increased risk of PS toxicity in the dark, its reduced photostability, shorter duration of the excited triplet, as well as higher production costs. The synthesis of a photosensitizer without heavy atoms is possible thanks to the use of electron donor–acceptor system technology in the spin–orbit charge-transfer intersystem crossing (SOCT-ISC) process. Currently, the main substrates for the synthesis of such photosensitizers are derivatives of the dipyrromethene boron dye (BODIPY) and naphthalimide derivatives in which the oxygen atoms are replaced with sulfur, thus achieving more effective production of free oxygen radicals even in poorly oxygenated tissue and negligible toxicity in the dark. In turn, the use of morphyl groups improved the selective accumulation of the photosensitizer in the lysosomes of cancer cells. These features make the above photosensitizers promising candidates for further testing, ultimately leading to trials for clinical use. In addition, it is worth noting the clinical aspects of the application of photodynamic therapy: proper training of staff and ensuring patient education about the nature of the treatment and possible side effects, so that they can make an informed decision about the treatment method. Moreover, PDT is still an alternative to standard treatments for cancer or skin lesions, performed in a limited number of centers, so its availability is limited. It is necessary to further interest the scientific community in the possibilities offered by modern technologies in the synthesis of new photosensitizers. The scientific works mentioned many times in this article prove that many of the newly designed compounds have great potential for practical use in PDT. However, it takes time for the obtained results to be confirmed in subsequent in vitro and in vivo studies and for some of them to finally be accepted for clinical testing. This process is necessary to evaluate the effectiveness of PDT in oncology or dermatology, and thus, to increase its availability.

## Figures and Tables

**Table 1:** Groups (generations) of synthetic photosensitizers, their representatives and their use in therapy.

PS Generation	Representative Compounds and the Activation Wavelength Range/Absorption Peak	Structure of the Molecule	Applications	References
I	Photofrin, 630 nm	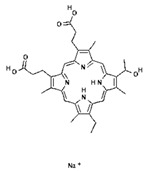	Cancers of the esophagus, lungs and bronchi	[16,57]
II	Ameluz 635 nm	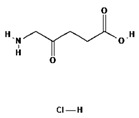	Basal cell carcinoma of the skin and actinic keratosis (Ameluz)	[16,58,59]
II	5-ALA, 630 nm	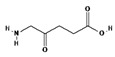	Imaging of brain tumors	[16,17,60]
II	HAL/Hexvix 380–450 nm	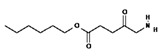	Bladder cancer	[16,61]
II	Metvix, 570 to 670 nm	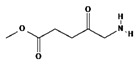	Basal cell carcinoma, Bowen’s disease and actinic keratosis	[16,59,62]
II	Foscan, 652 nm	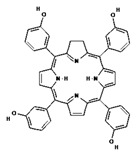	Head and neck cancer	[16,17,59,63]
II	Laserphyrin, 664 nm	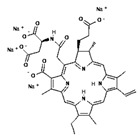	Esophageal cancer, lung cancer and brain tumors	[16,17,59,64]
II	Redaporfin, 749 nm	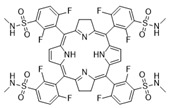	Cancer of the bile ducts	[16,17,59,65]
III	Conjugates of a photosensitizer with a plasma protein (hemoglobin or albumin molecule)	-	Under investigation	[66]
III	Nanocomplexes of cationic micelle, photosensitizer and small interfering RNA (siRNA)	-	Under investigation	[67]
III	Nanoconjugates of UCNs and photosensitizer, made from a nanoparticle of manganese dioxide (MnO_2_) and a biopolymer of hyaluronic acid (HA)	-	Under investigation	[68]
III	Organometallic Hf-porphyrin: DBP-UiO	-	Under investigation	[69]
III	Pluronic redaporphyrin micelles	-	Under investigation	[70]
III	Polymer nanoparticles on a monocytic carrier	-	Under investigation	[71]

**Table 2:** Other chlorin derivatives and their molecular structure.

Selected Chlorin Derivative	Molecular Structure	References
Chlorin p6	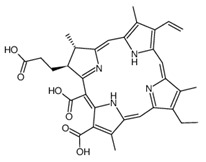	[75,77]
Purpurin	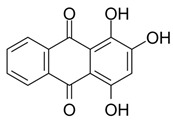	[75,78]
Photochlor	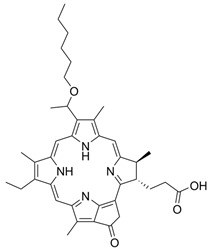	[75,79]
